# Resilience of Community Food Systems (CFS): Co-Design as a Long-Term Viable Pathway to Face Crises in Neglected Territories?

**DOI:** 10.3390/foods10030521

**Published:** 2021-03-02

**Authors:** Ana Paula Dias Turetta, Michelle Bonatti, Stefan Sieber

**Affiliations:** 1 Brazilian Agricultural Research Corporation (EMBRAPA Soils), Rio de Janeiro 22460-000, Brazil; ana.turetta@embrapa.br; 2 Program of Territorial Development and Public Policy, Federal Rural University of Rio de Janeiro, Seropedica 23890-000, Brazil; 3 Leibniz Centre for Agricultural Landscape Research (ZALF), Eberswalder Straße 84, 15374 Müncheberg, Germany; stefan.sieber@zalf.de; 4 Department of Agricultural Economics, Faculty of Life Sciences Thaer-Institute, Humboldt-Universität zu Berlin, Unter den Linden 6, 10099 Berlin, Germany

**Keywords:** community-based strategies, food security, socioecological innovations, stakeholders’ engagement, social learning, sustainability

## Abstract

The COVID-19 pandemic has brought on a global crisis, with impacts an ongoing food security and nutrition, exposing the vulnerabilities of our society. However, it can be a time for reflection and an opportunity to propose and stimulate initiatives that are ready to facilitate resilience within the food system. The food to fork must be shortened and diversified where it is viable and feasible, while made affordable for all societal levels. To face these challengers, the community food systems (CFS) approach has a crucial role, since it copes with relevant principles, including the necessities of low-income societies from areas particularly marginalized from mainstream food systems, of which those land areas also can pose as additional insurance just in case of occurrence of whatever crises. Systematizing the components and contributions of CFS can facilitate the advance of strategies to better deal with crises and increase resilience. Therefore, in this paper, through key elements of CFS, we propose a theoretical framework that can be applied by decision makers as a conceptual guide for combating threats to food systems in neglected territories.

## 1. Introduction

The COVID-19 pandemic exposed the world to unprecedented scenarios in our recent history, exposing the vulnerabilities of our society. One of the first impacts revealed was the running to supermarkets to guarantee food and basic supplies for an unknown number of coming days, causing a wave of stocking-up. Therefore, the first clash to food systems was related to food access. However, the passing days also reveal a crisis of planning and production in the agricultural process, threatened by labor shortages and poor harvests, imposing a severe test for our current food supply systems.

Surely the impact on food supply systems affects countries and societies differently. For many low-income countries, the long-term economic consequences will be more devastating than the disease itself, with coping strategies coming at the expense of such essential services as health and education [1].

Food exports have grown sixfold since 1990; four-fifths of people live, in part, on calories produced in another country [2]. This means that countries with a high dependency on food imports plus instabilities in their economic sector will struggle to mobilize enough resources to respond to crises.

Thus, the consequences of food-price spikes in low-income countries can be devastating and have severe long-term repercussions. The 2008 food-price crisis shows that suffering disproportionately affected the poorest households, often female-headed and with a high dependency ratio, as well as casual laborers and petty traders—rising food prices often increase the depth of poverty rather than pushing more people into poverty. Also hit hard were people living in remote areas, migrant and informal sector workers, people in humanitarian crises and conflict areas, and other vulnerable groups [3]. Food security implications are dire and may also imply subsequent crises such as riots and violence [3].

In short, the COVID-19 pandemic exposed the two main constraints of food systems. First, especially the globalized food systems are facing challenges such as the lack of stock in retail at the same time that the food production remains without access to channels of distribution. Second, the strong food systems concentration affected directly the neglected population that is the one already most severely hit by the crisis, contributing to the worsening of poverty and malnutrition [4].

Our food systems have been proven inefficient to fight key global problematics: hunger, obesity, and food loss [5]. This has been largely debated. However, other failures of our food systems (as social exclusion) have to be urgently included in the research–policy debate. Beyond all the measures that governments can take to reduce the risks to the population, it is urgent to consider food systems on the world security agenda, in order to minimize these dependencies and vulnerabilities where possible, while increasing transparency and traceability. This new way of thinking about food systems also supports the challenge of meeting population growth in light of the threats of climate change. Therefore, a new productivity revolution is required, considering all aspects of holistic food systems, engaging stakeholders as key actors in a trans- and interdisciplinary way, promoting agri-innovations in a co-design process, and considering actual costs having factored in all external effects including risks of sudden events such as pandemics. This is an opportunity to propose and stimulate initiatives that are able to facilitate resilience within the food system. The food to fork must be shortened and diversified where it is viable and feasible, while made affordable for all societal levels. The economic viability stays here as a challenge to be achieved, at the same time taking into account the high risk of pandemic costs, which are usually not included into food prices over a longer period (with a number of pandemic or similar events including climate change).

The community food systems (CFS) approach finds room in this discussion, since it copes with relevant principles, including the needs of low-income societies from areas particularly marginalized from mainstream food systems, of which those land areas can also pose as additional insurance in case of occurrence of whatever crises. It seeks to build up community food resources (supermarkets, farmers’ markets, gardens, transportation, community-based food processing ventures, and urban farms) to meet community needs; to incentivize self-reliance, empowering people to provide for their own food needs; and to promote local agriculture to build better links between farmers and consumers [6]. Adopting a more systemic perspective centered in communities’ power encourages a radical rethinking of how resilience of food systems can be re-conceptualized and practiced across global to local scales.

Systematizing the components and contributions of CFS can facilitate the development of strategies to better deal with crises and increase resilience. Therefore, in this paper, through key elements of CFS, we propose a theoretical framework that can be used by decision makers as a conceptual guide for combating threats to food systems in neglected territories.

## 2. Community Food Systems

The concept of community food security is first mentioned in 1994 as a response to the globalized food system [6]. In 1999, Biehler et al. [7] state that local efforts are an important driving force for the community food security movement, since it advocates for developing comprehensive community-based solutions for food systems problems.

One factor that can contribute to this lack of relation between local/cultural food systems and sustainability is the separation of the various institutions dealing with food (including relevant up- and downstream private sector corporations) that can lead to policies that are fragmented if not contradictory [7]. This lack of coordination considering multi-scale levels makes it difficult to piece together the puzzle of food-related policies and to identify weakness, barriers, or opportunities to advance community food systems. Simple maximization of normatively envisaged impacts is in practical terms irresolvable. A coalition of relevant food community systems stakeholders would benefit and improve the sustainability of local initiatives, since it can promote community engagement through empowerment; identify (local) demands; establish progressive producer outreach and new product and production development; develop appropriate infrastructure and resources for sustaining a community food system; work with regional economic development entities and local governments to give local agriculture a higher priority, visibility, and sustainability; promote transparency and traceability by installing a monitoring system that includes indicator progress reports and a database; and prepare a business model for food-based entrepreneurs to assist them in accessing small business technical assistance and resources [8].

Anderson and Cook [9] state that clear articulation within a theoretical framework is needed for community food security to be effective as a guide for policy and action. Lastly, a sustainably financed investment plan to cover all arisen costs need to be designed in the way that costs and benefits are in relative terms evenly distributed among all actors of the food system (no misuse of market power).

Thus, our proposal aims to fill this gap, presenting a theoretical framework for CFS to aid decision making processes in neglected territories, improving community nutrition quality and resilience in the face of global crises, like COVID-19. In our proposal, we understand as “neglected areas” those areas characterized mostly by the illegal occupation of land and high demographic density, informal settlements together with a notable lack of infrastructure and public services. These areas can exist in urban, peri-urban, or rural areas and are strongly present in low income countries.

One of the most important contributions of our CFS framework is to promote a shift from “neglected areas” to “reference areas of development and sustainability.”

**Figure 1:**
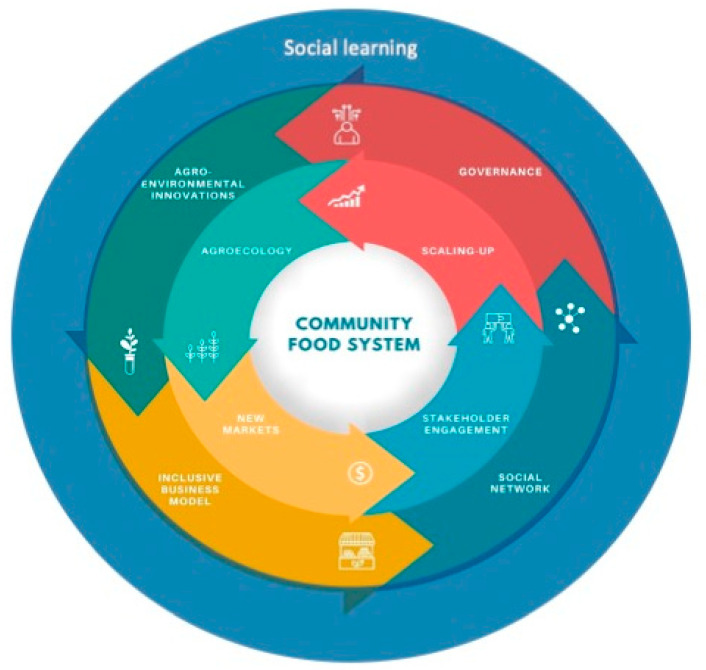
Linkages among the four CFS framework axes.

In our proposed framework (Figure 1), we highlight four main axes to achieve this goal:

- agro-environmental innovations, able to improve CFS resilience and upgrade population nutrition;
- inclusive business models that promote the connection between producers and consumers (economic autonomy in the community);
- reinforcement of social networks as social capital to create CFS opportunities;
- governance, which is a core aspect of our vision to address all the challenges in question; and
- social learning process as a crucial and transversal component that potentializes all the other components.

We present this framework as an alternative to strengthen CFS not only through food production, but also by creating a connection between processing, distribution, consumption, and waste management. Although a manifold framework does exist here, we want to emphasize the complementarity, linking the holistic re-consideration of social capital and governance in combination of innovative business-based approaches.

Agroecology innovations, new markets, stakeholder engagement, and scaling-up are the proposed linkages among the axes (Figure 1).

### 2.1. Agro-Environmental Innovation

Elias and Marsh [10] consider that sustainability innovations in agricultural and food systems are situated within three broader concepts: socio ecological systems, integrated landscape management, and the rural–urban interface.

The agroecology concept, through its 10 elements, covers all of these aspects and comprises all the elements necessary to optimize the CFS. The 10 elements of agroecology seeking a smooth and sustainable transition along with the diversification of production sources are diversity, synergies, efficiency, resilience, recycling, co-creation and sharing of knowledge, human and social values, culture and food traditions, responsible governance, and circular and solidarity economy [11].

Agroecology considers agriculture in its multifaceted aspects, including its core aspect to make the whole food system more sustainable and resilient, balancing the trade-offs inherent to this activity. Improving multifunctionality can range from enhanced agricultural practices and nutrition, while ensuring low carbon dioxide emissions and allowing for a climate resilient economy [8].

Pretty [12], comparing the impacts of 286 agroecology projects, finds that such interventions increased productivity on 12.6 million farms, with an average crop. The improvement is also verified in the ecosystem services provision, such as carbon (C) balance (factoring in all costs of externalities).

Agroecological diversification strengthens ecological and socio-economic resilience by offering a variety of products, eligible to access new market opportunities. Successful platforms typically blend innovative marketing and financing with the scaling-up of farm- based agroecological practices to meet landscape objectives [10]. Complementary information and communication technologies (ICT) technology can be used to lower costs and enhance information asymmetries for higher market transparency.

### 2.2. Inclusive Business Model (IBM)

FAO defines IBM “as a form of internal organization of an initiative that can effectively integrate smallholders into markets—either as producers or consumers—with the underlying assumption that there are mutual benefits for both smallholders and the private sector” [13]. This means that the main idea of IBM is a business model that is embedded in communities, such that the benefits reach producers, consumers, and intermediaries alike.

The most important market channels for agroecological products are already identified: direct sales and on-farm sales, farmers’ markets and eco-fairs, as well as restaurants and hotels. The most important benefits from selling/buying directly through a variety of direct sales mechanisms are related to social relationships such as proximity, conviviality, and trust [13]. Additionally, Eufemia et al. [14] state that strengthening associative and cooperative models (associativism) can improve the role of economic models at the community level.

A renewed interest in direct sales from farmers to customers with various new innovations is increasing. Community supported agriculture, food circle buying clubs, seasonal food box subscriptions, and on-line farm shops are ongoing experiments. Benefits include reduced packaging, improved product freshness and transparency, shorter supply chains, and developing relationships between farmers and consumers [15].

Nevertheless, the COVID-19 outbreak exposed new faces of food consumption. Global food e-commerce sales that were forecast to nearly triple by 2023, rising to $321 billion and accounting for nearly 5% of total e-commerce revenues, will probably increase even further, since people are choosing to get their food from e-commerce outlets [16]. Therefore, IBM can be seen as complementary measure to the global scale, which operates at low-cost level with short transport distances and cooperative-based community systems.

However, it is evident that, especially in neglected territories and low-income countries, there are many facilities improvements through investments, such as internet access and data security policies as well as capacity building, that are necessary steps to access this new market. Cost sharing models may help for viable IBMs.

Another well-informed challenge for agroecological products market access is related to transport issues (logistics) and the lack of widespread consumer awareness [17]. Challenges at regional/local level of transport and infrastructure need to be solved here. For example, inefficient transportation and logistics can be responsible for most of the production waste generation [15]. There is no one solution to address this issue. Making food value chains even shorter (in case of inefficiency) or more resource efficient as such can help to reduce waste, but it is still necessary to improve the connection between producers and consumers, carefully identifying the choke points and tailoring solutions.

A key aspect to win and expand the market for agroecological products is consumer awareness. Most initiatives report that intermediaries and consumers lack information about agroecological products and production practices; thus, they are highly influenced by untrustworthy or incorrect information about the safety and price of these products [17]. Nevertheless, in interviews with agroecological consumers from different countries, 74% of respondents report that the main reason for joining the initiative was an interest in improving their health [17]. This is a clear indication that there is a room in creating value for agroecological quality and increasing trust in agroecology products. A prerequisite that must be set to become embedded in new markets is investments in traceability and certification processes. It can be also a strategy to improve food safety, since foodborne diseases represent a barrier to smallholder farmers to access high value domestic markets [17].

There is evidence about strategies to contact and direct communication between consumers and producers (through social media, the internet, personal exchanges, farm visits, and ICTs via smart phone applications, etc.) as well as quality control systems that enable producers to communicate quality through certification and labels. Labels are important as a means to communicate agroecological quality and the main reason for adopting a label is to create an identity for producers (brand label) or for their vision of agroecology (differentiated label) [18].

### 2.3. Social Networks

Silici [19] highlights that the adoption of agroecological practices requires access to skills and information, strengthening of local knowledge, incremental learning, and links to social networks. Aiming at facilitating the exchange of experiences and co-creation processes as social capital of the community, by promoting the stakeholder dialogues, local producers, regional–national networks/alliances, and governments are allied. A core concept in this process is social networks analysis that can set a clear distinction between the notion of markets and transactions in terms of exchange of products, labor, and services [20]. Once stakeholder engagement is strengthened, it is possible to raise the voice of local governments in the regional/national arena.

The analysis of social networks is fundamental for understanding food systems, since they are characterized and affected by webs of complex interactions—cutting across multi-level borders—and feedback loops, broad constellations of policies, as well as multi-scale power relations and the political economy [20].

These analyses can capture the complexity of emerging challenges through the mapping and analysis of the interplay of actors with the economic, social, environmental, and natural resources related food systems issues. It allows researchers to zoom in on individual behaviors (micro-level) within a system, on the sub-systems (meso-level), and on the whole network properties (macro-level) [20].

Lee et al. [21] state that larger, older, and better-connected communities contribute more to the household food security of their members, as well as that participation in rural livelihood activities may have spill-over benefits, by promoting food security at the community level. In contrast, households in newly formed communities are at disproportionate risk of food insecurity, despite factors such as smaller household sizes, greater education, and per-capita incomes similar to those of households in older communities.

Understanding the linkages between actors and strengthening stakeholder engagement are prerequisites for improving the inclusiveness and governance efficiency of food systems, because they allow actors to establish strategic links between institutions to pursue individual or collective goals.

### 2.4. Governance

Governance models offer valid tools to resolve conflicts and engage different stakeholders. These also help to promote the community perspective, where inclusion and commitment are key factors [22].

Our framework considers the Community-Based Governance (CBG) approach, which is a bottom-up organizational model [14]. The CBG approach aims to increase the participation of local groups in the planning, research, development, management, and formulation of policies and strategies for a wider community. The attention and inclusion of local perspectives allow for a synthesis of collective problems and the development of joint solutions to solve them [14]. Successful experiences demonstrate that once producers, processors, and consumers are highly involved in the governance process, it is easy to promote the community leverage [18].

The stakeholder engagement is a pre-requisite to scaling up community initiatives and can efficiently increase the socioeconomic impact from a small to a large scale of coverage [23]. Global experience with scaling up community development projects shows that strong political commitment to decentralization and empowerment is essential, and that a local champion often leads the process [24]. However, the same authors highlight that often politicians and bureaucrats oppose, or at least do not support, shifting power to the grassroots.

Thus, governance is a key topic in scaling up transition. Hartmann and Linn [24] state that the larger the number of sectors involved in project interventions, the more complex the scaling up. This highlights the relevance of the circular aspect of the proposed CBF framework, which requires knowledge of the social–political–environmental–economic reality and a co-creation process promoted by community engagement. Combined, these aspects have the potential to generate highly focused initiatives, which are relevant for a successful scaling-up process.

### 2.5. Social Learning

Much has to be understood about how community food systems can be better implemented. In this sense, social learning is crucial to achieving all the elements proposed. Social learning presents itself as a transversal element that embeds and potentializes all other components (Figure 1). Learning spaces among all value chain actors should be promoted (through consumer education campaigns, advanced training about agroecology, and business models at the community level, etc.). The mobilization and integration of different stakeholders, such as technical staff (to analyze the agriculture systems and markets, propose improvements, etc.), practitioners, producers, and consumers, is necessary for reinforcing community engagement and collective knowledge development.

## 3. Recommendations

The COVID-19 outbreak is exposing the fragility of the actual global food system. This is the main motivation for seeking new food models that are able to improve the resilience of food-systems and food safety, shortening the field–fork distances. We believe that CSF is a feasible solution to reach food systems justice and alleviate poverty and malnutrition, especially in neglected territories where the negative impact of the crises can be more harmful.

Hence, we propose a feasible circular framework that connects well-established concepts with alternatives for their implementation. This connectivity addresses broad aspects of food systems, reinforcing those linkages that meet multiple goals, scales, and sectors. It allows multiple stakeholders to sketch out a range of pathway solutions and highlight trade-offs. It can also be connected to national objectives and global targets, including climate and biodiversity international agreements, and this strategy can also link the food culture with sustainability, since the community will express its experiences and have the opportunity to improve it through technical knowledge.

There is no time-frame for the CFS framework implementation, since it depends on different communities’ characteristics, such as size, social engagement and social network, availability of food system analysis (production, accessibility, nutrition, markets), natural characteristics, and facilities available, among others. However, we suggest the social network analysis as the first framework step, since it is critical for having a good picture of community structures and functioning, a key aspect for the upcoming axis.

## Data Availability

Not applicable.
